# Green and Rapid Preparation of Fluorosilicone Rubber Foam Materials with Tunable Chemical Resistance for Efficient Oil–Water Separation

**DOI:** 10.3390/polym14081628

**Published:** 2022-04-18

**Authors:** Wan-Jun Hu, Qiao-Qi Xia, Hong-Tao Pan, Hai-Yang Chen, Yong-Xiang Qu, Zuan-Yu Chen, Guo-Dong Zhang, Li Zhao, Li-Xiu Gong, Chang-Guo Xue, Long-Cheng Tang

**Affiliations:** 1 College of Material, Chemistry and Chemical Engineering, Key Laboratory of Organosilicon Chemistry and Material Technology of Ministry of Education, Hangzhou Normal University, Hangzhou 311121, China; ahczhwj@163.com (W.-J.H.); xtdfst199409@163.com (Q.-Q.X.); 2020111017016@stu.hznu.edu.cn (H.-T.P.); 2021111017009@stu.hznu.edu.cn (H.-Y.C.); yxqu11@163.com (Y.-X.Q.); czy18867809793@163.com (Z.-Y.C.); lizhao@hznu.edu.cn (L.Z.); anily_love@163.com (L.-X.G.); 2 School of Material Science and Engineering, Anhui University of Science and Technology, Huainan 232001, China; chgxue@foxmail.com; 3 Key Laboratory of Silicone Materials Technology of Zhejiang Province, Hangzhou Normal University, Hangzhou 311121, China

**Keywords:** fluorosilicone rubber foam, room-temperature foaming, mechanical flexibility, chemical resistance, oil–water separation

## Abstract

Polydimethylsiloxane (PDMS) foam materials with lightweight, excellent oil resistance and mechanical flexibility are highly needed for various practical applications in aerospace, transportation, and oil/water separation. However, traditional PDMS foam materials usually present poor chemical resistance and easily swell in various solvents, which greatly limits their potential application. Herein, novel fluorosilicone rubber foam (FSiRF) materials with different contents of trifluoropropyl lateral groups were designed and fabricated by a green (no solvents used) and rapid (<10 min foaming process) foaming/crosslinking approach at ambient temperature. Typically, vinyl-terminated poly(dimethyl-co-methyltrifluoropropyl) siloxanes with different fluorine contents of 0–50 mol% were obtained through ring-opening polymerization to effectively adjust the chemical resistance of the FSiRFs. Notably, the optimized FSiRF samples exhibit lightweight (~0.25 g/cm^−3^), excellent hydrophobicity/oleophilicity (WCA > 120°), reliable mechanical flexibility (complete recovery ability after stretching of 130% strain or compressing of >60%), and improved chemical resistance and structural stability in various solvents, making them promising candidates for efficient and continuous oil–water separation. This work provides an innovative concept to design and prepare advanced fluorosilicone rubber foam materials with excellent chemical resistance for potential oil–water separation application.

## 1. Introduction

Silicone rubber foam (SiRF) is one of the most versatile porous polymeric foam materials, which has been widely used in the fields of transportation, electronics, aerospace, and national defense because of its light weight, good mechanical flexibility, and facile processability [1,2,3,4,5,6,7]. The molecular structure of polydimethylsiloxane (PDMS) foam, as the most representative one of SiRF materials, is composed of a crosslinked –Si–O–Si– backbone and many −CH_3_ pendant moieties. The structural features make it possess intriguing properties, such as wide temperature usage range (−60–220 °C), mechanical flexibility, high thermal and chemical resistance, and thermal insulating performance [8,9]. Therefore, the PDMS foam material has drawn considerable academic and industrial attention during the past few years [10,11,12,13,14,15]. With the ever-increasing development of society, the demand for polymer foam materials at home and abroad continues to grow [16,17,18,19,20,21,22] and more demands are imposed on the production and performance of all polymeric foams, particularly for SiRFs. Unfortunately, the −CH_3_ pendant moiety of PDMS-based SiRF has low polarity [23] and the solubility parameter (δ) of PDMS (δ = 7.3 (cal/cm^3^)^1/2^) [24] is similar to that for nonpolar organic solvents such as hexane (δ = 7.2 (cal/cm^3^)^1/2^). It is easy to absorb solvent and swell upon exposure to the most nonpolar or low-polar environment, resulting in deformation and even failure of PDMS foam structural components, which seriously limits its application [25,26], especially in the field of oil–water separation application [27].

To overcome the above drawbacks, many strategies have been developed to enhance the solvent resistance of the PDMS foam materials. Generally, the modifying strategies of the PDMS materials could be divided into physical and chemical methods. Physical modification was usually accomplished by coating some hybrid inorganic/organic polymers or addition of various fillers so that the material’s swelling ratio to organic solvent could be reduced. The chemical method usually refers to grafting some functional groups to the PDMS chains [28,29]. For instance, Peng et al. used oxygen plasma and trichloro(1H,1H,2H,2H-perfluorooctyl) silane to surface-modify the PDMS foam materials that were prepared by dissolved sugar method [30], and the modified sample presented excellent hydrophobicity. However, these above strategies still show some shortages, e.g., the unchanged intrinsic chemical resistance of the PDMS foam materials, the uncertain structure stability of filler/polymer coatings in organic solvents, and impractical toxic solvents used. Moreover, most of the above approaches still need lengthy and complex steps, e.g., centrifugation and vacuum, high temperature drying, and complex special facilities. Therefore, it is urgent and challenging to develop an extremely simple, green, and rapid approach to prepare silicone rubber foam materials with tunable chemical resistance.

As is well-known, the fluorine-containing groups (e.g., −CH_2_CH_2_CF_3_) with high polarity could reduce the material’s surface energy, and they thus are usually used to incorporate into the polysiloxanes to improve the material’s chemical resistance in both nonpolar solvents and oils [31]. Previous studies have demonstrated that the chemical resistance of traditional silicone rubber could be enhanced by the partial replacement of methyl groups with some fluorine groups [32]; for example, fluorosilicone rubber [33,34,35]. However, there are few reports about the preparation of fluorosilicone rubber foam materials. Recently, Métivier et al. used supercritical CO_2_ as the foaming agent to prepare poly(methylvinyldimethyl)siloxane/fluorosilicone foam materials via physical foaming method [36]. Unfortunately, the density of the fluorosilicone rubber foam materials was more than 0.50 g/cm^3^, demonstrating the closed-cell structure, which cannot be used for oil–water separation. Therefore, developing an extremely simple, green, and facile methodology for fabricating novel, lightweight, mechanically flexible, and chemical-resistant fluorosilicone rubber foam (FSiRF) materials is still of great significance.

In this work, we designed and synthesized vinyl-terminated poly(dimethyl-co-methyltrifluoropropyl) siloxanes with different fluorine contents via anionic ring-opening polymerization. Water was used as an effective foaming agent to prepare the novel fluorosilicone rubber foam materials through a simple chemical dehydrogenative foaming method under ambient temperature condition. The foaming process can be finished in only several minutes and no solvents were used, showing unique features such as environment-friendliness, safety, facile, and no residual foaming agent. The prepared FSiRF samples show good compatibility with hydrogen dimethicone, and the fluorine content could be controlled by simply adjusting the composition of the starting compounds. By the partial replacement of methyl groups with some trifluoropropyl groups, the solvent and oil resistance of FSiRF samples are greatly improved in comparison with traditional PDMS foam materials; the porous FSiRFs feature high compressibility and stretchability, outstanding hydrophobicity/oleophilicity, as well as high structural stability. Discrepancies in the pore structure, mechanical properties, thermal stability, and solvent resistance of the five FSiRFs with different fluorine content were investigated. Moreover, the solvent-resistant mechanisms were also discussed and the application of the material in oil–water separation is also demonstrated.

## 2. Experimental Section

### 2.1. Materials

1,3,5-Tris(3,3,3-trifluoropropyl)-1,3,5-trimethylcyclotrisiloxane (D_3_^F^) was provided by Shandong Weihai Xinyuan Chemical Co., Ltd. (Weihai, China). Octamethylcyclotetrasiloxane (D_4_) and hydrogen dimethicone (PDMS-H, 1.6 wt.% hydrogen group) was supplied by Zhejiang Xin’an Chemical Industrial Group Co., Ltd. (Hangzhou, China). Vinyl dimethicone with low viscosity values (MM^Vi^) was supplied by Zhejiang Runhe Silicone New Material Co., Ltd. (Ningbo, China). Tetramethylammonium hydroxide (TMAH, as catalyst for the preparation of vinyl-terminated poly(dimethyl-co-methyltrifluoropropyl) siloxane), was provided by Sinopharm Group Co., Ltd. (Shanghai, China). Karstedt’s catalyst, diluted to a concentration of 3000 ppm, was supplied by Betely Polymer Materials Co., Ltd. (Suzhou, China). D_3_^F^ and D_4_ were vacuum-dried before use. Other materials were directly used without any further purification.

### 2.2. Preparation of PDFS-Vi-X

PDFS-Vi-X (Vinyl-terminated poly(dimethyl-co-methyltrifluoropropyl)siloxane) with varying fluorine content (where X represents the methyltrifluoropropylsiloxane unit ratio) is synthesized via anionic ring-opening copolymerization (AROP) of cyclic oligomers [37]. The specific synthesis steps are as follows: a certain amount of D_4_, MM^Vi^, and TMAH were added into a three-neck flask with a stir paddle and condenser under nitrogen atmosphere. The mixture was stirred at 90 °C and dropped D_3_^F^ into the flask with a constant pressure dropping funnel. After dropping D_3_^F^, the solution was heated to 110 °C for 4 h, then to 150 °C for 30 min to decompose the catalyst TMAH. The decomposing products of TMAH (mainly trimethylamine, methanol) unreacted D_4_ and D_3_^F^, and low-molecular-weight products were removed at 170 °C in vacuum and the viscous PDFS-Vi-X were obtained. The feeding number of reactants to synthesize PDFS-Vi-X is listed in Table S2.

The molecular weight values and polymer dispersity index (PDI) of PDFS-Vi-X are listed in Table 1.

### 2.3. Preparation of FSiRF Materials

According to the previous study [11], the preparation of FSiRF materials includes the following steps. First, a prepolymer blend was prepared by mixing the 0.2-g inhibitor, 10-g PDMS-H, and a certain content of water (0.2–1.8 wt%, as foaming agent) at a speed of ~1200 rpm for 5 min to achieve a uniform blend. Next, the PDFS-Vi-X (70 g) and 4 g Karstedt’s catalyst was dispersed in the above blend and stirred at 1200 rpm for 30 s. Afterwards, the above blend was poured into a mold and foamed at ambient temperature for ~8 min. Finally, the sample was cured at 100 °C for 2 h in an oven to obtain the FSiRF-Y (where Y means FSiRF materials prepared with different PDFS-Vi-X; for example, FSiRF-1 corresponds to PDFS-Vi-12.5%, FSiRF-2 corresponds to PDFS-Vi-25.0%) materials.

### 2.4. Characterizations

Fourier transform infrared spectra (FTIR) of PDFS-Vi-X were carried out using FTIR spectroscopy (Nicolet 5700, Thermo Scientific, Waltham, MA, USA) in the range of 600–4000 cm^−1^ using the KBr pellet technique. ^1^H NMR spectra of PDFS-Vi-X were measured by a Bruker AV400MHz spectrometer (Bruker, Karlsure, Germany) with deuteroacetone (CD_3_COCD_3_) as the solvent. The number-average molecular weight (Mn) was gauged by gel permeation chromatography (GPC) on a Waters Breeze instrument (Waters, Milford, MA, USA) by using THF as the eluent (1 mL/min) and a Waters 2410 refractive index detector (Waters, Milford, MA, USA).

The foaming process of prepolymer blend sample was observed by optical microscopy (Eclipse LV00POL, Nikon, Tokyo, Japan). The structure and morphology of FSiRF were examined using scanning electron microscopy (SEM) (Sigma-500, ZEISS, Oberkochen, Germany). Thermogravimetric analysis (TGA) and derivative thermogravimetric analysis (DTG) were performed on a PerkinElmer Pyris Thermogravimetric Analyzer (TA-Q500, TA Instruments-Waters LLC, Newcastle, DE, USA) using 5–10 mg of samples. The experiments were performed at a heating rate of 10 °C·min^−1^ in air from 35 to 800 °C. The compressive characteristics of the samples were tested by a DMA (TA-Q800, TA Instruments-Waters LLC, Newcastle, DE, USA) at a strain rate of 2000 μm·min^−1^. Water contact angle (WCA) of various samples were measured with a DSA30 CA analyzer (Kruss, Hamburg, Germany) using a 3 μL water droplet, and the reported results are the average value of five parallel measurements.

The swelling properties of FSiRF samples were measured using a homemade swelling measurement system that utilizes a Panasonic HG-C1050 micro laser distance sensor (HG-C1050, Panasonic, Osaka, Japan) to gauge the displacement of a detector in contact with the foam sample, thus producing the transient and steady-state change in height of the sample as it swells or shrinks. The instrument is capable of measuring swelling ratios with a position resolution of ±10 μm. The samples in this study are found to be isotropic, and the volume change can be easily determined. The swelling ratio (Q) was calculated as follows:

$$Q=\frac{v_{2}}{v_{1}}$$

where V_1_ and V_2_ are the volumes of the specimens before and after the tests.

## 3. Results and Discussion

### 3.1. Preparation and Performance of FSiRF Materials

**Figure 1:**
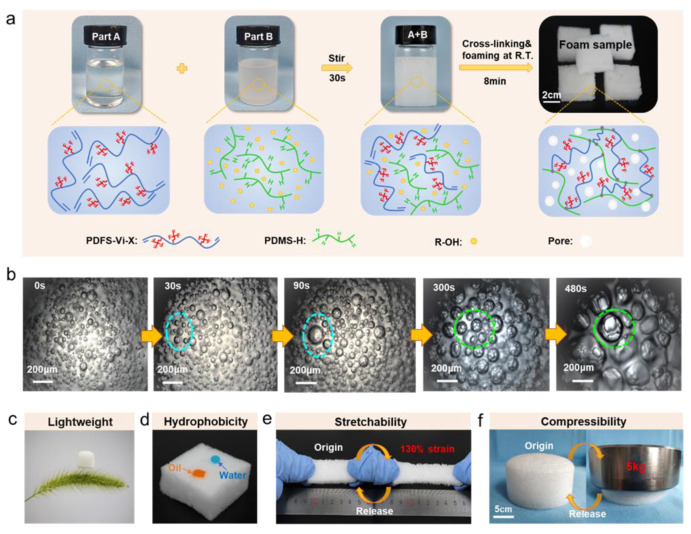
(**a**) Schematic illustration of the fabrication process of FSiRF materials. (**b**) Optical microscopy image of PDFS-Vi-X/PDMS-H mixture before and during foaming process. (**c**) Typical image of FSiRF sample on a foxtail grass without bending any hairy branches, indicating a lightweight nature of FSiRF materials. (**d**) FSiRF materials with a drop of water (blue color) and oil (orange color) on the surface, showing strong oleophilicity and high hydrophobicity of the surface. Digital images of FSiRF materials under (**e**) a stretching and releasing cycle and (**f**) a compressing and releasing cycle, showing good reversible stretchability and compressibility.

Figure 1a shows the schematic illustration of the fabricating process of FSiRF materials, which includes a rapid foaming and crosslinking process at room temperature. Typically, the inhibitor, PDMS-H, and a certain content of water (as foaming agent) are mechanically mixed to obtain a highly dispersed suspension; then, the PDFS-Vi-X and Pt catalyst were added into the above suspension to prepare the FSiRF materials. After that, the above mixture can react at room temperature to crosslink and generate hydrogen gas; the detailed reactions will be discussed later. Using optical microscopy, we found that the water foaming agent is phase-separated in the PDFS-Vi-X/PDMS-H mixture due to their different polar properties, but these agents also present as highly dispersed in the mixture. With increasing time, more and more bubbles were generated in the matrix (Figure 1b). Notably, these bubbles can grow or combine during the foaming process in a few minutes, thus forming a porous silicone rubber foam structure with pore size of several hundreds of micrometers.

It is worth noting that the prepared FSiRF sample is so light that a piece of sample with a size of 20 mm × 20 mm × 20 mm could stand on the top of a foxtail grass without bending any hairy branches, as shown in Figure 1c. The density values of FSiRF samples were measured and are 0.24–0.25 g/cm^3^, as shown in Figure S1. Meanwhile, the prepared FSiRF sample displays excellent surface hydrophobicity and oleophilicity. When a water droplet (blue color) is placed on the surface of the FSiRF material, the contact angle reveals high hydrophobicity (~130°) (see Figure 1d and Figure S2) with neither physical nor chemical surface treatment. The good hydrophobicity could be owing to a combination of microporous morphological structures and the low surface energy of fluorosilicone molecules. While a drop of oil (orange color) was put on the surface of the FSiRF, and it was immediately absorbed into the foam, resulting in a contact angle of nearly 0° (Figure 1d). The rapid absorption of the oil can be mainly owed to the strong oleophilic nature of the FSiRF and its microporous features, which can induce capillary action [36]. More significantly, the FSiRF material possesses intriguing mechanical elasticity, which can be stretched to 130% strain or compressed to about 60% strain without breaking apart and can completely restore to its original shape (Figure 1e,f). Such excellent stretching and compressing performance without any deformation are rarely observed in other polymer foams and aerogel materials (e.g., inherently brittle silica aerogels) with high porosity [37,38].

### 3.2. Molecular Design and Synthesis of PDFS-Vi-X

**Figure 2:**
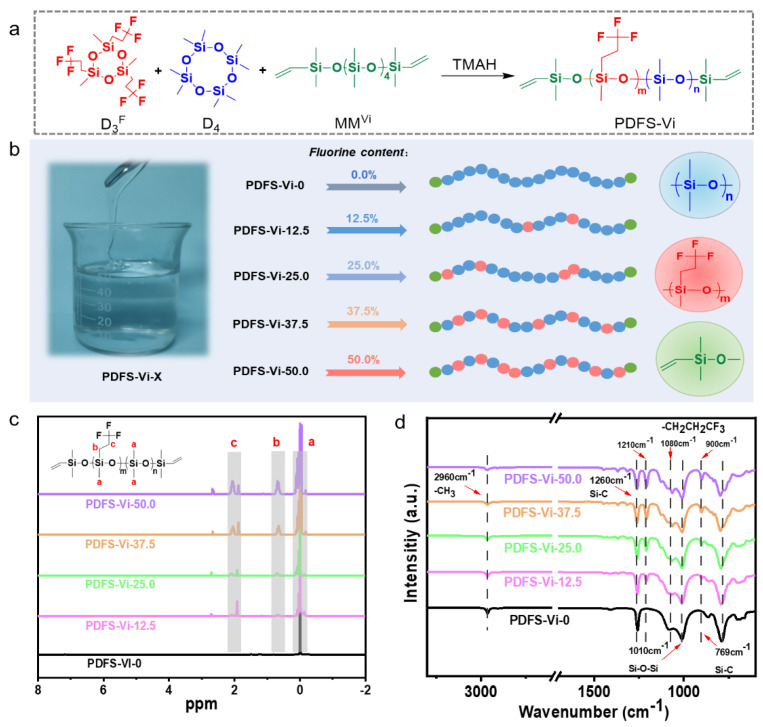
(**a**) Chemical synthesis of PDFS-Vi synthesis process. (**b**) Schematic illustration of the fabrication process of PDFS-Vi-X with different fluorine contents. (**c**) ^1^H NMR spectra and (**d**) FTIR spectra of PDFS-Vi-X.

To meet the high controllability requirements of the molecular structure of the silicone foam material, a series of PDFS-Vi-X molecules were designed and synthesized, and the basic synthesis process was shown in Figure 2a. Typically, D_3_^F^ and D_4_ can react via a ring-opening polymerization, and the MM^Vi^ was used to block the above reaction, thus obtaining the PDFS-Vi prepolymer. Clearly, the PDFS-Vi-X with different molar contents of pendant trifluoropropyl groups were synthesized by simply altering the ratio of D_4_ and D_3_^F^ and anionic polymerization time. Figure 2b illustrates that the five typical molar contents (0, 12.5%, 25.0%, 37.5%, and 50.0%) of trifluoropropyl groups can be successfully regulated by changing the molar ratio of D_4_ to D_3_^F^ [38], and the molecular weight (M_n_, M_w_, and PDI values) shown in Table 1 suggests a relatively good distribution of molecular weight of the synthesized PDFS-VI-X molecules, which is helpful for the preparation of the FSiRF materials.

The successful chemical synthesis of PDFS-Vi-X was evidenced by the ^1^H NMR and FTIR spectra (Figure 2c,d). The structure of PDFS-Vi-X copolymers with different fluorine contents can be evidenced through ^1^H NMR tests (Figure 2c) by the appearance of four peaks at 0.1, 0.75, 2.0, and 5.8 ppm, which are owing to the chemical shifts of –Si–CH_3_, –CH_2_CH_2_–, –CH_2_CH_2_CF_3_, and –Si–CH = CH_2_, respectively, demonstrating the successful introduction of trifluoropropyl groups [39]. With the increase in fluorine content, the corresponding peaks gradually become stronger, confirming the effective tunability of trifluoropropyl groups in the PDFS-Vi-X chain. (The details of the ^1^H NMR spectrum of PDFS-Vi-X are shown in Figure S7.) Figure 2d shows the FTIR spectra of PDFS-Vi-X copolymers with different fluorine content. As expected, a characteristic peak at 2960 cm^−1^ was the signal of –CH_3_ bond, and the characteristic vibration absorptions at 1260 and 769 cm^−1^ represent the symmetrical deformation and stretching vibrations of the Si–C bond, respectively. Moreover, characteristic vibrations at 1010 cm^−1^ correspond to the stretching vibrations of Si–O–Si bond. The typical peaks at 1210, 1080, and 1720 cm^−1^ were associated with the asymmetrical deformation, symmetrical deformation, and stretching vibrations of the –CH_2_CH_2_CF_3_, respectively. It is worth noting that the intensity of these three characteristic peaks become stronger with increasing fluorine content of the PDFS-Vi-X copolymers. The related viscosity and yield of the synthesized PDFS-Vi-X are shown in Figure S3, and the results indicate the relatively high yield (>80%) of the target molecules. The above results confirm the successful synthesis of the PDFS-Vi-X copolymers with tunable molecular structure, which agrees well with other studies [40,41].

### 3.3. Pore Microstructures of FSiRF Materials

**Figure 3:**
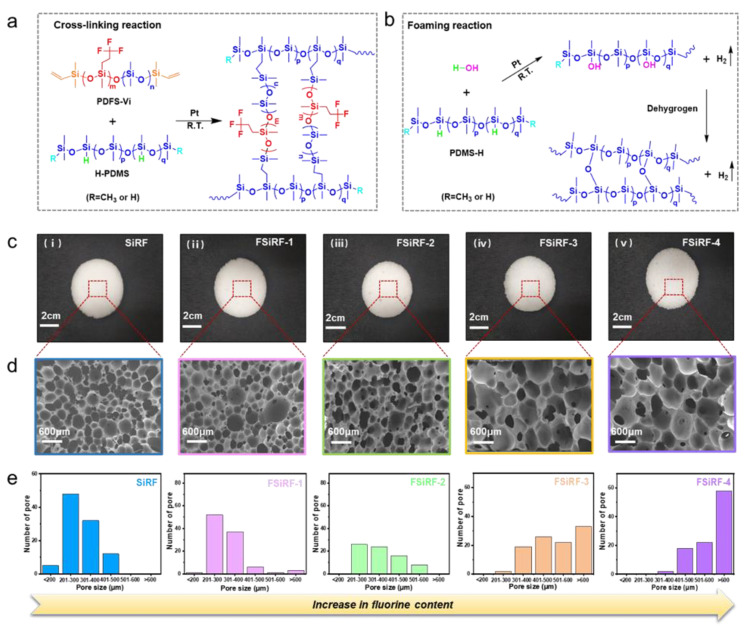
(**a**) Crosslinking and (**b**) foaming process of FSiRF materials at ambient temperature. Structural characterization and analysis of various FSiRF samples. (**c**) Photographs of (**i**) pure SiRF, (**ii**) FSiRF-1, (**iii**) FSiRF-2, (**iv**) FSiRF-3, and (**v**) FSiRF-4. (**d**) SEM micrographs and (**e**) pore size distribution of FSiRF materials with different fluorine contents, indicating the pore size increases with the increase in fluorine content.

The pore structure of the prepared FSiRF foams is determined by the effective balance of the crosslinking and foaming reactions during the fabricating process. Typically, the crosslinking reaction refers to the hydrosilylation reaction between hydrogen dimethicone molecules and vinyl-terminated poly(dimethyl-methyltrifluoropropyl)siloxane molecules under the Pt-based catalyst to form a polymer network (see Figure 3a). The foaming reaction means that the foaming agent (water) and hydrogen dimethicone polysiloxane react under Pt-based catalyst catalysis to release hydrogen to form a porous structure (see Figure 3b). We do know that many factors would affect the size and uniformity of the pore structure of the silicone rubber foam. Besides the dispersion of the foaming agent in the system, the most important factor is the balance of the reaction rates of foaming and crosslinking [42]. In the initial stage, the crosslinking reaction and foaming reaction are carried out at the same time. If the crosslinking degree of the system is not sufficient and the viscosity is too low, the generated bubbles are easy to migrate in the system and, thus, form some large bubbles. With the progress of the crosslinking reaction, the crosslinking degree of the system increases, the viscosity also increases, and the gas produced by the foaming reaction is hard to migrate in the system. After completion of the foaming reaction and crosslinking reaction, the pore structure was finally formed. When the fluorine content is low, the terminal vinyl of PDFS-Vi-X behaves with high reactivity [43]. With increasing fluorine content, the activity of terminal vinyl reduces intensely due to the steric hindrance of the trifluoropropyl group, which would affect the pore morphology and structure.

To evaluate the fluorine content on influencing the pore structure, the five kinds of FSiRFs with different pore structures were designed, and the digital images, SEM images, and pore size distribution of these FSiRF samples with different pore sizes were shown in Figure 3c–e. Clearly, the pore size of FSiRF samples is strongly dependent on the content of the trifluoropropyl groups. These FSiRF materials with different pore sizes have a comparatively complete porous structure, and the pore size changes from less than 200 μm to greater than 600 μm. It can be seen in Figure 3c that the prepared FSiRFs are white, and the size distribution of the pores is uniform. Two different kinds of porous structure were shown in SEM micrographs (Figure 3d): pure SiRF foam (without fluorine) presents spherical cells, while the other FSiRFs present ellipsoid shape. The peak of cell size distribution in Figure 3e indicates that increasing fluorine content obviously raises the pore size of the foam; as fluorine content varies from 0 to 50%, pore size increases from 200–300 μm to greater than 600 μm (Figure 3e). Moreover, the distribution of pore size also becomes narrower with increasing pore size, and the uniformity of the cellular structure gradually decreases.

### 3.4. Mechanical and Thermal Properties of FSiRF Materials

**Figure 4:**
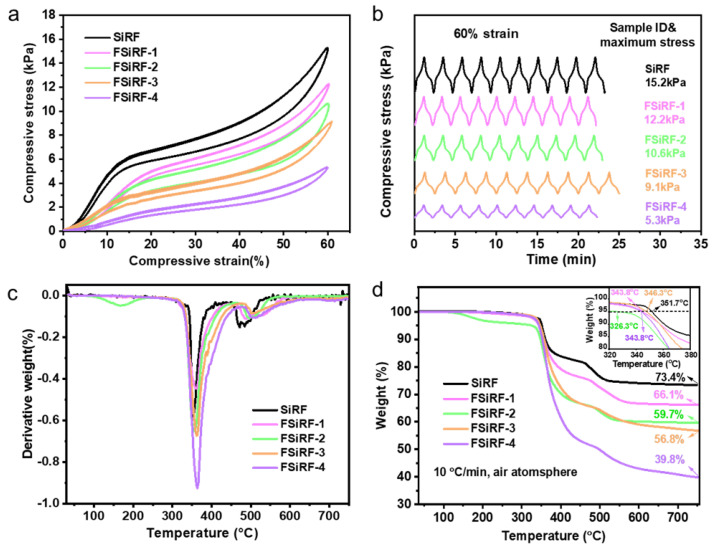
Mechanical and thermal properties of FSiRF samples. (**a**) Compressive stress−strain curves of the FSiRF materials with different fluorine content at strain = 60%, and (**b**) compression cycle tests at strain = 60% of various FSiRF samples, demonstrating the mechanical stability of the materials. (**c**) TGA and (**d**) DTG curves of FSiRF materials under air conditions.

Figure 4 shows the mechanical and thermal properties of the FSiRF materials. The stress–strain curves of the FSiRFs with different fluorine content is shown in Figure 4a. As can be seen, uniaxial compressive tests display an evident drop in the maximum stress values at strain = 60% with increasing fluorine content compared with that of pure SiRF materials, e.g., ~9.1 kPa for the FSiRF-3 and ~5.3 kPa for the FSiRF-4, which is inferior to that of pure SiRF with ~15.2 kPa. Normally, a higher ratio of the open-cell structure generates a much lower value in the maximum stress value [44]. As the fluorine content increases, the crosslinking reaction rate gets slower, and the open-cell content becomes larger. This is the most important factor leading to a lower compressive stress value of the FSiRF materials. In Figure 4b, the compressive strength of the FSiRFs remains almost unchanged after 10 compression cycles, which shows the excellent mechanical flexibility and reliability of FSiRFs.

The TGA and DTG curves of SiRF and FSiRF materials under air conditions from room temperature to 750 °C are measured and shown in Figure 4c,d. Pure SiRF starts to degrade at about 351.7 °C, owing to the thermal pyrolysis of pendant groups, and retains a steady weight residue of above 520 °C. Evidently, with the increase in fluorine content, the thermal stability of RSiRF composite foams significantly declined; for instance, the decomposition temperature at 5 wt% weight loss of FSiRF-4 decreases by ~8 °C (see Figure 4c and Table S1) compared with pure SiRF. In addition, the remaining weight at 750 °C of SiRF is also reduced after the addition of trifluoropropyl groups, e.g., from 73.4 wt% for pure SiRF to 56.8 wt% for FSiRF-3 and 39.8 wt% for FSiRF-4 sample, respectively. As the temperature rises, the trifluoropropyl groups of the side chain degrade at first, and the molecular weight of the trifluoropropyl group is larger than that of the methyl group [45]. As a result, the mass loss of the FSiRF under high temperature conditions is larger than that of pure SiRF. Figure 4d reveals that the degradation curves have two stages, and the relative parameters are shown in Table S1. The first stage ranges from 350 °C to 400 °C. The mass loss at this stage raises with increasing trifluoropropyl content [46]. This stage corresponds to the decomposition of these side chain organic groups. The second stage ranges from 450 °C to 500 °C, which is related to the decomposition of main Si–O–Si chains. Of course, the degradation processes under air atmosphere are complex, and the concrete mechanism of thermal pyrolysis needs to be further researched.

### 3.5. Chemical Resistance of FSiRF Materials

**Figure 5:**
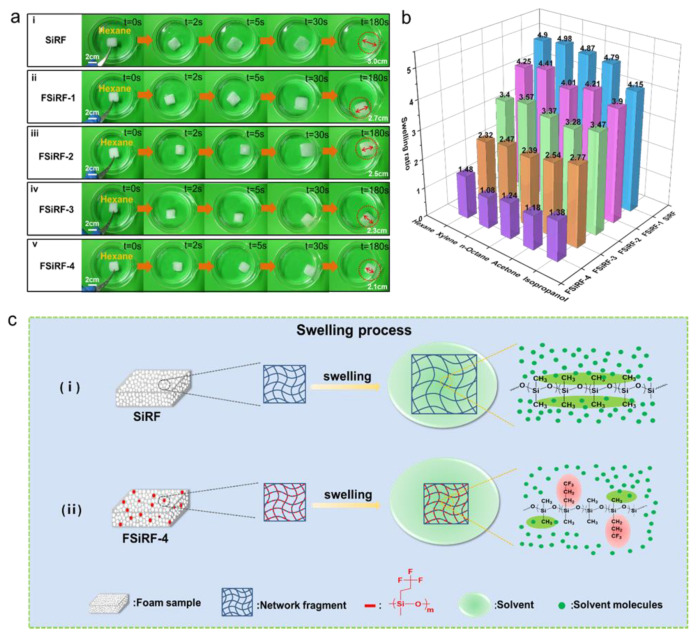
(**a**) Swelling process of the FSiRF materials in Hexane and (**b**) swelling ratios of FSiRF materials for different organic solvents, which shows that the swelling resistance of FSiRF material is enhanced with the increase in fluorine content. (**c**) Diagram and comparison of the antiswelling mechanism of FSiRF materials with different fluorine contents of 0 and 50%.

The swelling phenomenon of polymer foam materials is an obstacle to the promising application in oil–water separation. To analyze the swelling ratio of the FSiRF materials, the samples were immersed in different solvents and chemicals, e.g., n-hexane, xylene, n-octane, acetone, and isopropanol. Figure 5a shows the representative swelling process of the FSiRFs in n-hexane. It can be clearly observed that when SiRF is put into n-hexane, its volume expands rapidly until reaching saturation equilibrium. Normally, when the foam is immersed in the solvent, the solvent molecules quickly spread into the crosslinking network of the foam; thus, the volume of the foam expands. Consequently, the contractile force of the internal crosslinking network increases gradually. When the contractile force is equal to the force of solvent diffusion, the swelling equilibrium and the volume no longer change [47]. Notably, with the increase in fluorine content, the foams show better resistance to organic solvents and the swelling volume is smaller, indicating good structural stability. Figure S4 displays the digital photographs of the FSiRF samples with different fluorine contents before and after swelling in n-hexane. Using the equilibrium swelling method to test the swelling capacity of FSiRF materials, the swelling degree is calculated by measuring the size change in real time. The results are shown in Figure S5; the FSiRF foams undergo swelling and equilibrium processes. With increasing fluoride content, the swelling capacity of FSiRFs decreased and the swelling resistance of the FSiRF sample was obviously improved.

In comparison with methyl-vinyl silicone rubber foam, the oil and solvent resistance of fluorosilicone rubber foam is obviously prominent and can be tuned [48]. We investigated the swelling of FSiRF materials and analyzed the effect of the content of trifluoropropyl groups on the chemical resistance of fluorosilicone rubber foam in the following solvents (i.e., n-hexane, xylene, n-octane, acetone, and isopropanol). Each sample was immersed in the solvents at room temperature for 24 h. The volume changes of each sample swelled in the solvents were recorded. The swelling ratio (Q) is defined as follows:

$$Q=\frac{V_{2}}{V_{1}}$$

where V_1_ and V_2_ are the volumes of the samples before and after the tests. Figure 5b shows swelling ratios of FSiRF materials in the above organic solvents and the specific information is listed in Table 2. As we can see from the data, the swelling ratios of foams with more content of trifluoropropyl groups in solvent are much smaller than those of pure SiRF material. For example, the swelling ratio from ~4.41 in xylene for 12.5 mol% to ~1.08 for 50 mol% trifluoropropyl groups, which is inferior to that (~4.98) of the PDMS foam materials with a lateral methyl group. The swelling ratio ranges from ~3.28 in acetone for 25.0 mol% to ~1.18 for 50 mol% trifluoropropyl groups, which is inferior to that (~4.79) of the PDMS foam materials with a lateral methyl group. Figure 5b shows that with the increase in fluorine content, the swelling ratio of foam in organic solvents decreases gradually, which proves that the introduction of fluorine-containing groups can endow silicone rubber foam with excellent chemical resistance and swelling resistance. The higher the fluorine content is, the more obvious the swelling resistance is. Therefore, the chemical resistance of fluorosilicone rubber foam materials can be tuned by controlling the amount of trifluoropropyl groups introduced.

The mechanisms of tunable chemical resistance were proposed and illustrated in Figure 5c. When a crosslinked polymer contacts with a solvent, the network absorbs a certain amount of solvent, which is largely dependent on the polymer–solvent interactions [49,50]. As shown in Figure 5c, the polarity of dimethyl silicone rubber foam molecules is very small. According to the “Polarity nearness” rule in the polymer–solvent system, some nonpolar or weak-polar solvent molecules can infiltrate into the crosslinking network of SiRF and swell, leading to an increase in volume and a reduction of physical properties. The chemically stable trifluoropropyl groups as substituents can “shield” the main chain of Si-O-Si in fluorosilicon molecules and increase the polarity of the molecular chain, which improves the chemical resistance of FSiRF materials. The feature further protects against the infiltration of solvent molecules into the crosslinking network, and obviously improves the structural stability of silicone rubber foams in various solvents.

### 3.6. Oil–Water Separation of the FSiRF Materials

**Figure 6:**
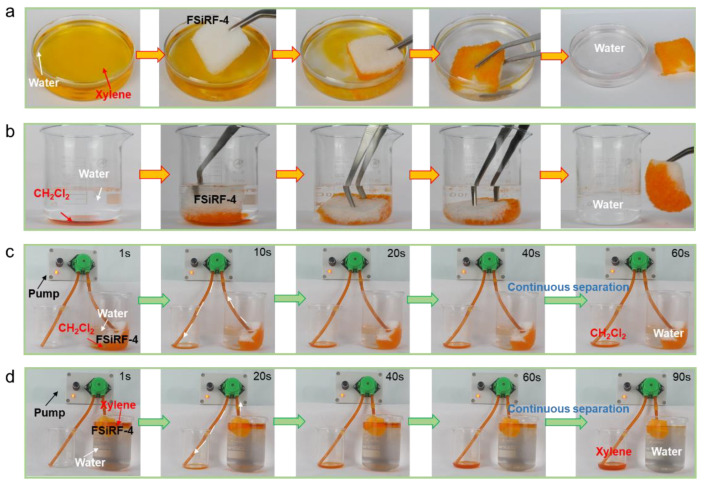
The performance of FSiRF-4 samples in the oil–water separation process. Removal of (**a**) light organic solvent (xylene dyed by orange) on water surface and (**b**) heavy organic solvent (dichloromethane dyed by orange) underwater via FSiRF-4 material, demonstrating good absorption capacity. The illustration of continuous oil–water separation performances of FSiRF-4 by plugging it with a pump, pumping (**c**) light xylene and (**d**) heavy CH_2_Cl_2_ from the oil–water mixture via FSiRF-4 samples. They passed through FSiRF-4 porous samples effectively and quickly, and no water went through the sample owing to the excellent surface hydrophobicity and oleophilicity of the samples.

By taking advantage of oleophilic and hydrophobic properties, good mechanical flexibility and reliability, as well as excellent tunable chemical resistance, the FSiRF material can be an ideal candidate as an efficient absorbent material that can absorb oil from oil–water mixtures. During the experiment, we found that the dimensional stability of the FSiRF materials is closely related to the separation performance. In this paper, the FSiRF-4 material with the best separation performance was selected as the demonstration for the application of oil–water separation. As shown in Figure 6a, the porous FSiRF-4 sample with the best chemical resistance is used to absorb the light organic solvent (xylene dyed by orange), which is floating on water. The xylene was entirely absorbed by FSiRF-4, thereby, it was eliminated from the water without any solvent left, confirming the good solvent absorption capacity. Moreover, the FSiRF-4 samples can also imbibe oils or organic solvents that are denser than water, such as dichloromethane (dyed by orange), which sinks under the water. We can see from Figure 6b that dichloromethane is immediately absorbed by the FSiRF-4 sample without wetting by water. As another proof, the FSiRF-4 is fixed in a syringe, and a mixed solution of water (dyed by blue) and chloroform (dyed with orange) is poured from the beaker. Chloroform immediately sinks and passes through the foam because of its larger density than water; however, water remains on the top surface of the foam. While a mixed solution of water (dyed by blue) and xylene (dyed with orange) is poured from the beaker, as xylene is lighter than water, the mixture remains on the top surface of the foam (Figure S6). These results indicate that the FSiRF-4 can be used for separating both light and heavy oil from water with selective absorption.

Except uncomplicated adsorption and extruding for oil absorption and recovery, we tried to continuously separate the oil–water blend under the assistance of a peristaltic pump. The separation process is as follows: one port of the rubber tube is plugged with FSiRF-4 while the other port is placed in a clean beaker, and the side containing the foam is put in an oil–water blend. Based on a simple combination of FSiRF-4 sample with pipes and a peristaltic pump to achieve continuous collection of oil from the oil–water mixture [51], the oil–solvent collection process is shown in Figure 6c–d. In the pumping process shown in Figure 6c, 50 mL heavy chloroform (dyed orange) can be immediately absorbed by the macropores of the FSiRF-4 material and then pumped into the clean beaker in 60 s. During the above processes, no water could pass through the FSiRF-4 due to the excellent surface hydrophobicity [52]. In Figure 6d, the floating n-hexane (dyed orange) can be collected from the water surface and pumped into the beaker in 90 s. After the above continuous oil–water separation process, the dimensions of the FSiRF-4 sample remained almost unchanged. (It shows that the structural stability of FSiRF-4 in continuous oil–water separation is better than that of PDMS foam. The comparison experiment is shown in Figure S8). Based on the results, it is clear that the optimized fluorosilicone foam materials with high trifluoropropyl content prepared in this work show promising application in the field of oil–water separation prospects.

## 4. Conclusions

In summary, we successfully synthesized vinyl-terminated poly(dimethyl-co-methyltrifluoropropyl) siloxanes with different trifluoropropyl contents of 0–50 mol% and prepared a series of FSiRF materials via a green, facial, and rapid foaming method under ambient temperature. The FSiRF samples feature light weight (0.24–0.25g/cm^−3^), excellent hydrophobicity/oleophilicity (WCA > 120°), and excellent mechanical flexibility and cyclic elasticity; and they can completely recover their original shape even after being stretched to 130% strain or compressed over 60%. Notably, with increasing fluorine content, the FSiRF materials display excellent structural stability and excellent solvent–oil resistance in various solvents (e.g., swelling ratio from ~4.41 in xylene for 12.5 mol% to ~1.08 for 50 mol% trifluoropropyl groups, which is inferior to that (~4.98) of the PDMS foam materials with a lateral methyl group. Further, the optimized FSiRF samples with 50 mol% trifluoropropyl groups demonstrated excellent adsorption potentiality for both floating or heavy solvent–oil and consecutive oil–water separating ability. Clearly, this work extends the design and development of novel high-performance fluorosilicone rubber foam materials with good mechanical flexibility (for both stretching and compressing) and tunable chemical resistance, showing promise for potential oil–water separation application.

## Figures and Tables

**Table 1:** Molecular weight values of PDFS-VI-X with different fluorine contents.

Sample Code	Fluorine Content (mol%)	M_n_ (g/mol)	M_w_ (g/mol)	PDI (M_w_/M_n_)
PDFS-Vi-0	0.0%	116833	153094	1.36
PDFS-Vi-12.5	12.5%	83139	116431	1.40
PDFS-Vi-25.0	25.0%	73677	103899	1.41
PDFS-Vi-37.5	37.5%	78373	105495	1.35
PDFS-Vi-50.0	50.0%	87643	122486	1.39

**Table 2:** Swelling ratios of FSiRF materials for different organic solvents.

Sample Code	Swelling Ratio (100%)				
	Hexane	Xylene	N-Octane	Acetone	Isopropanol
SiRF	4.90	4.98	4.87	4.79	4.15
FSiRF-1	4.25	4.41	4.01	4.21	3.90
FSiRF-2	3.40	3.57	3.37	3.28	3.47
FSiRF-3	2.32	2.47	2.39	2.54	2.77
FSiRF-4	1.48	1.08	1.24	1.18	1.38

## Data Availability

Not applicable.

## Supplementary Materials

The following supporting information can be downloaded at https://www.mdpi.com/article/10.3390/polym14081628/s1, Figure S1: The density of FSiRF materials with different fluorine contents; Figure S2: Surface water contact angles (insets are the water contact angles) of FSiRF materials; Figure S3: Viscosity and yield of PDFS-Vi-X with different fluorine contents; Figure S4: Photographs of FSiRF materials before and after swelling; Figure S5: Swelling capacity of FSiRF materials in hexane and xylene, respectively; Figure S6: Photographs of separated oil (dyed orange)/water (dyed blue) mixture for the FSiRF materials; Figure S7: Details of the 1H NMR spectrum of PDFS-Vi-X; Figure S8: The performance of SiRF samples in the oil/water separation process; Table S1: Detailed information of TGA curves under air conditions; Table S2. The feeding number of reactants to synthesize PDFS-Vi-X.
