# Supplementary material for: Green and Rapid Preparation of Fluorosilicone Rubber Foam Materials with Tunable Chemical Resistance for Efficient Oil–Water Separation
Source: Polymers (Basel). 2022 Apr 18;14(8):1628. doi: 10.3390/polym14081628 (PMC9028597; doi:10.3390/polym14081628)
Supplement: Supplementary file 1 [file polymers-14-01628-s001.zip › polymers-1669398-supplementary.pdf]

Supplementary Materials for

Green and Rapid Preparation of Fluorosilicone Rubber Foam Materials with Tunable Chemical Resistance for Efficient Oil–Water Separation

Wan-Jun Hu [1,†], Qiao-Qi Xia [1,†], Hong-Tao Pan [1], Hai-Yang Chen [1], Yong-Xiang Qu [1], Zuan-Yu Chen [1], Guo-Dong Zhang [1,*], Li Zhao [1], Li-Xiu Gong [1], Chang-Guo Xue [2], and Long-Cheng Tang [1,3,*]

[1] College of Material, Chemistry and Chemical Engineering, Key Laboratory of Organosilicon Chemistry and Material Technology of Ministry of Education, Hangzhou Normal University, Hangzhou 311121, China; ahczhwj@163.com (W.-J.H.); xtdfst199409@163.com (Q.-Q.X.); 2020111017016@stu.hznu.edu.cn (H.-T.P.); 2021111017009@stu.hznu.edu.cn (H.-Y.C.); yxqu11@163.com (Y.-X.Q.); czy18867809793@163.com (Z.-Y.C.); lizhao@hznu.edu.cn (L.Z.); anily_love@163.com (L.-X.G.)

[2] School of Material Science and Engineering, Anhui University of Science and Technology, Anhui, Huainan 232001, China; chgxue@foxmail.com

[3] Key Laboratory of Silicone Materials Technology of Zhejiang Province, Hangzhou Normal University, Hangzhou 311121, China

* Correspondence: zhangguodong@hznu.edu.cn (G.-D.Z); lctang@hznu.edu.cn (L.-C.T)

† These authors contributed equally to this work.

This file includes:

Figs. S1 to S7

Table. S1 to S2

Figure S1. The density of FSiRF materials with different fluorine contents.

Figure S2. Surface water contact angles (insets are the water contact angles) of FSiRF materials.

Figure S3. Viscosity and yield of PDFS-Vi-X with different fluorine contents.

Figure S4. Photographs of FSiRF materials before and after swelling.

Figure S5. Swelling capacity of FSiRF materials in hexane and xylene, respectively.

Figure S6. Photographs of separated oil (dyed orange)/water (dyed blue) mixture for the FSiRF materials.

Figure S7. Details of the ^{1}H NMR spectrum of PDFS-Vi-X.

Figure S8. The performance of SiRF samples in the oil/water separation process.

Table S1. Detailed information of TGA curves under air conditions.

Table S2. The feeding number of reactants to synthesize PDFS-Vi-X.

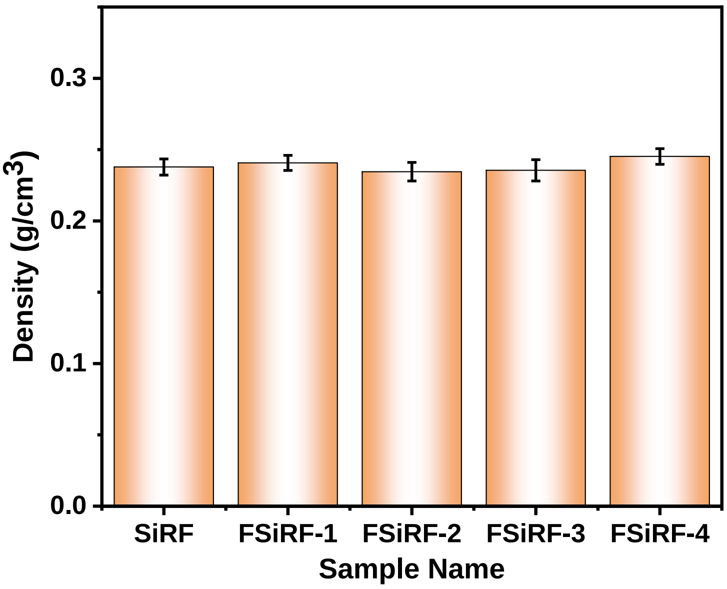

Figure S1. The density of FSiRF materials with different fluorine contents.

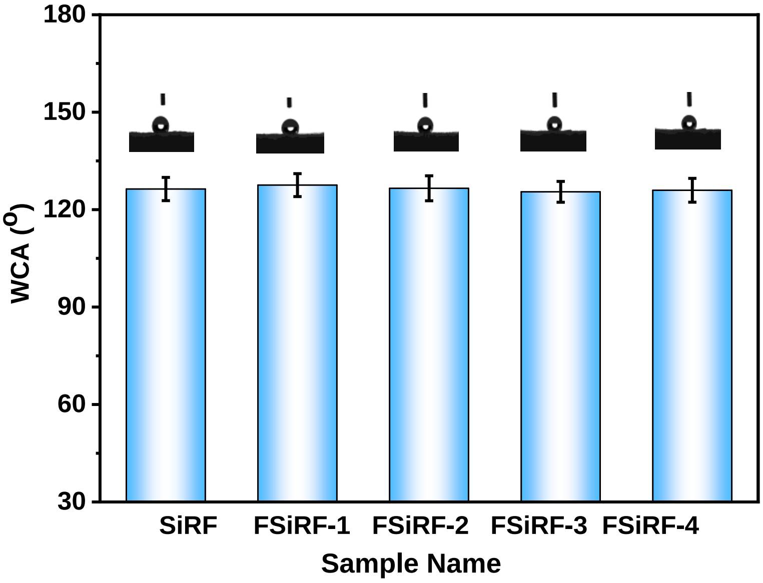

Figure S2. Surface water contact angles (insets are the water contact angles) of FSiRF materials.

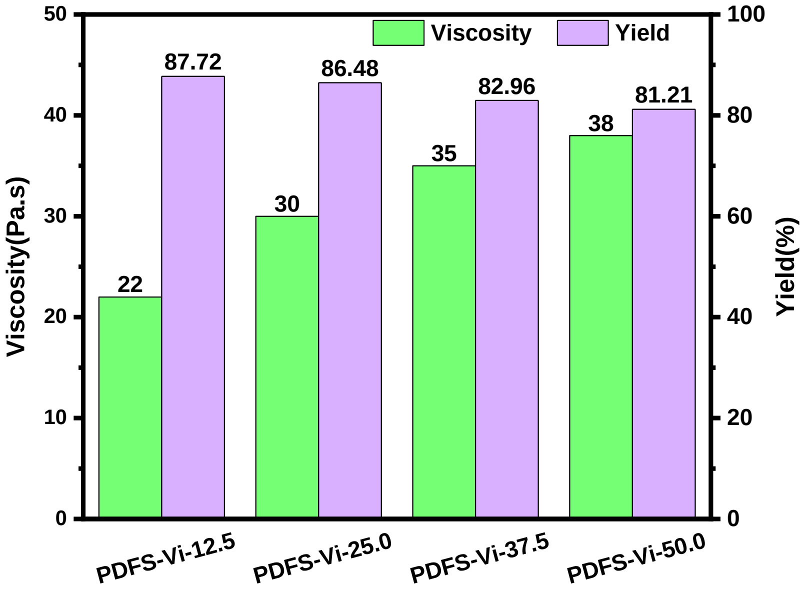

Figure S3. Viscosity and yield of PDFS-Vi-X with different fluorine contents.

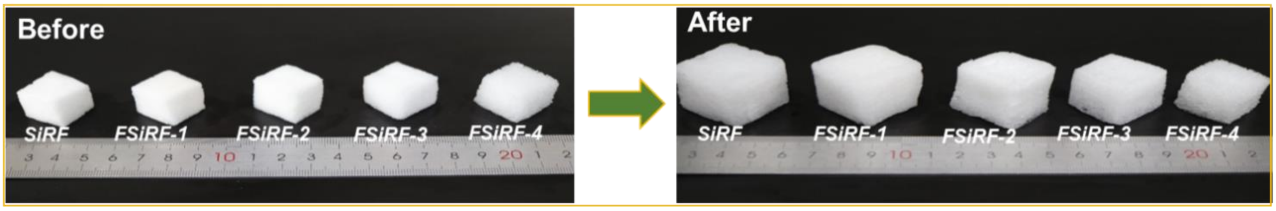

Figure S4. Photographs of FSiRF materials before and after swelling.

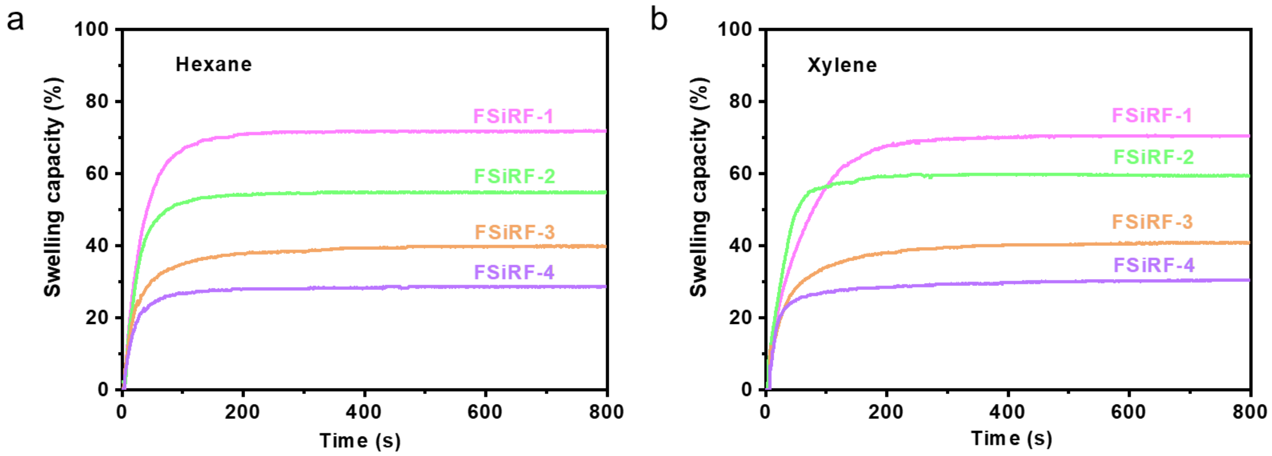

Figure S5. Swelling capacity of FSiRF materials in hexane and xylene, respectively.

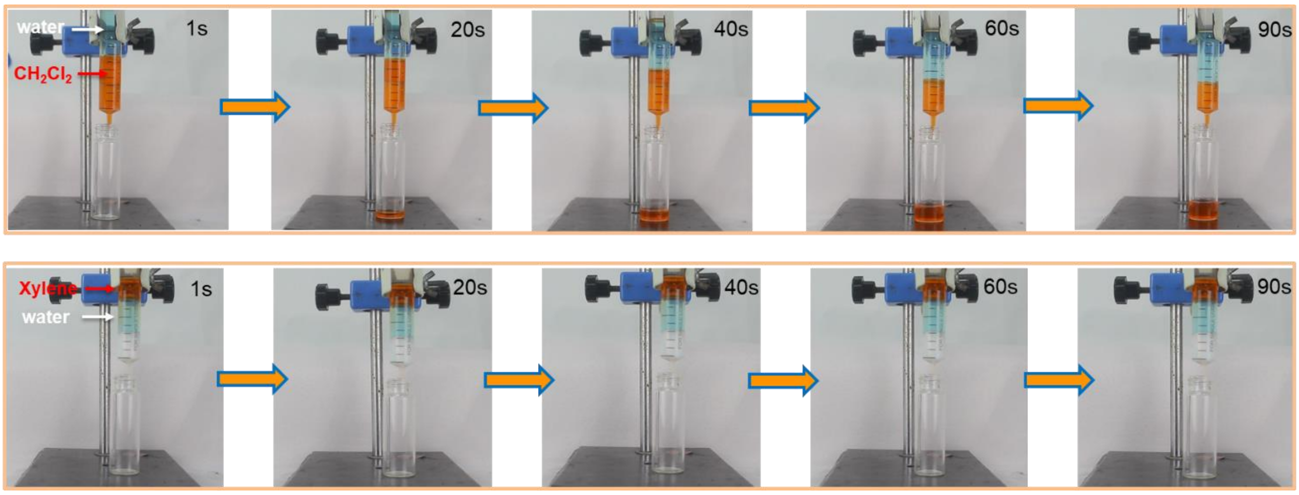

Figure S6. Photographs of separated oil (dyed orange)/water (dyed blue) mixture for the FSiRF materials.

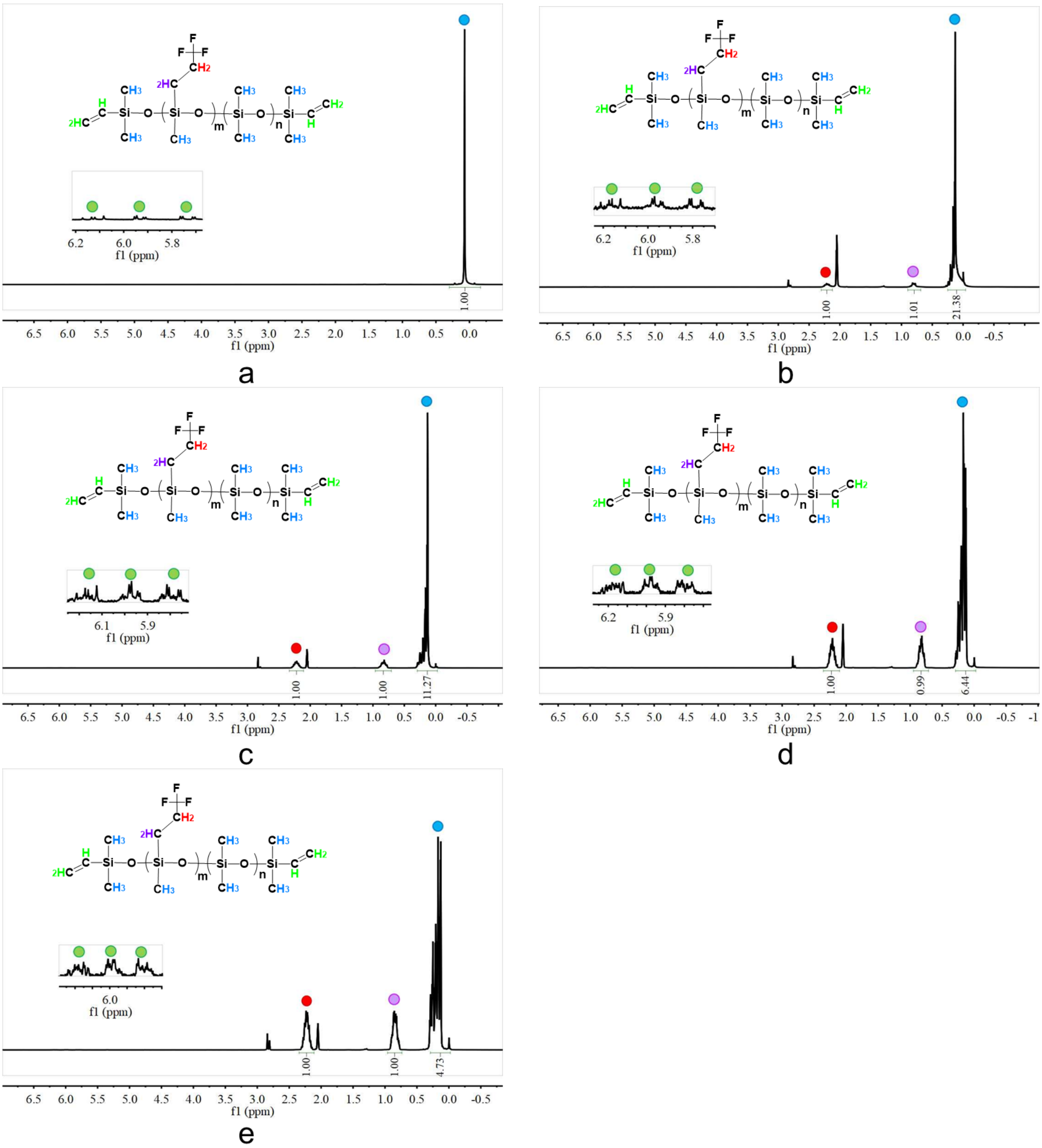

Figure S7. Details of the ^{1}H NMR spectrum of PDFS-Vi-X. ^{1}H NMR spectrum (400 MHz) of (a) PDFS-Vi-0 in $CDCl_3$, (b) PDFS-Vi-12.5 in CD_3COCD_3, (c) PDFS-Vi-25.0 in CD_3COCD_3, (d) PDFS-Vi-37.5 in CD_3COCD_3, (e) PDFS-Vi-50.0 in CD_3COCD_3.

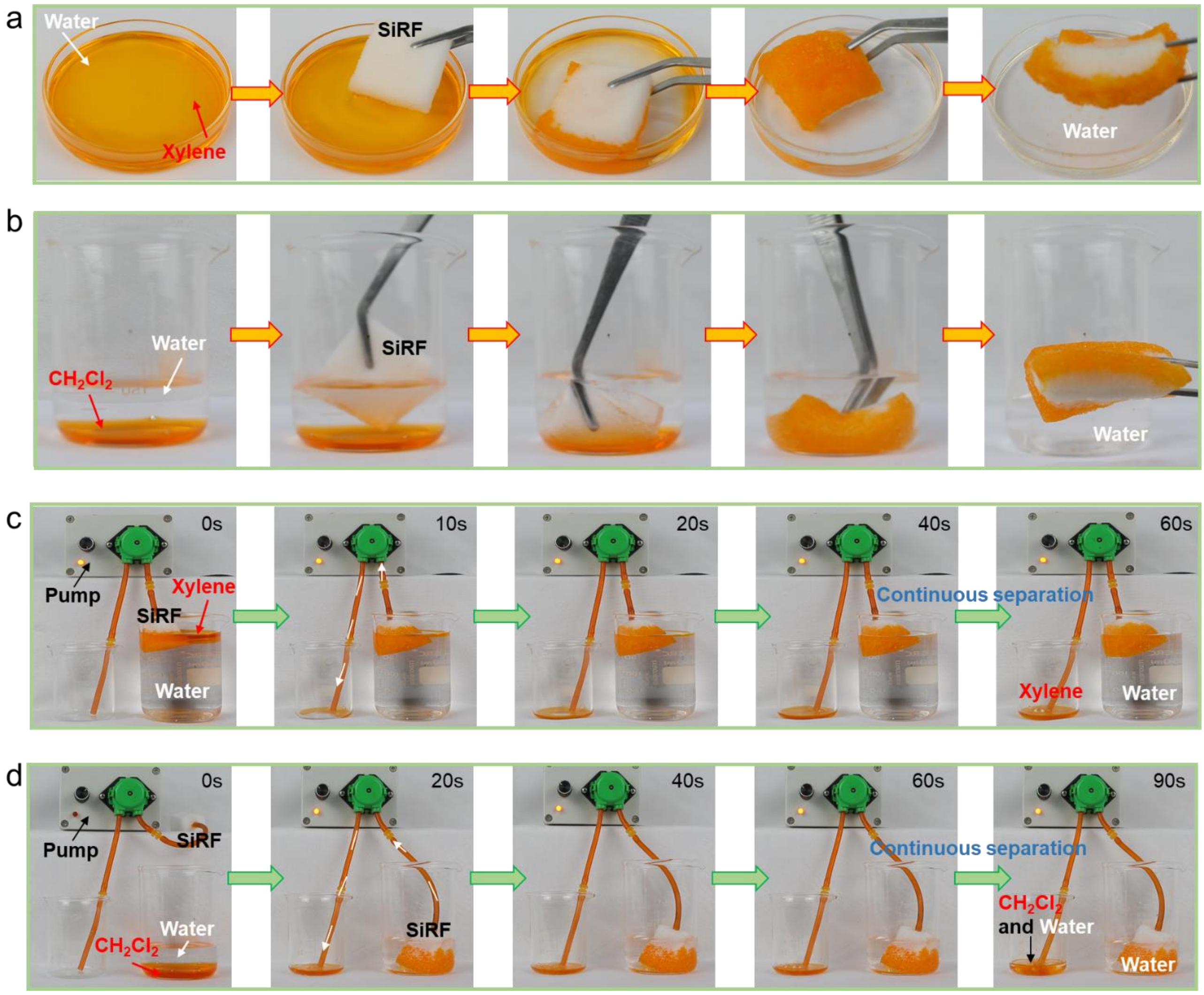

Figure S8. The performance of SiRF samples in the oil/water separation process. Removal of (a) light organic solvent (xylene dyed by orange) on water surface and (b) heavy organic solvent (dichloromethane dyed by orange) underwater via SiRF material. The SiRF material with poor dimensional stability swells evidently after absorbing organic solvent. The illustration of continuous oil/water separation performances of SiRF by plugging it with a pump, pumping (c) light xylene and (d) heavy CH_2Cl_2 from oil/water mixture via SiRF samples. Due to SiRF material is extremely swellable, its continuous oil/water separation performance is very poor compared to FSiRF-4. In (d), there is even water passing through the SiRF material (obvious phase separation can be seen).

Table S1. Detailed information of TGA curves under air conditions.

Sample code	$T_{5wt\%}$ (℃)	$T_{10wt\%}$ (℃)	$T_{20wt\%}$ (℃)	Residue at 750℃ (%)
SiRF	351.7	361.7	471.7	73.4
FSiRF-1	343.8	356.3	371.5	66.1
FSiRF-2	326.3	348.8	363.8	59.7
FSiRF-3	346.3	356.3	373.8	56.8
FSiRF-4	343.8	351.3	364.0	39.8

Table S2. The feeding number of reactants to synthesize PDFS-Vi-X.

Sample code	Fluorine content (mol%)	D_4 (g)	D_3^F (g)	MM^{Vi} (g)	Catalyst (ppm)
PDFS-Vi-0	0.0%	296	0	4	25
PDFS-Vi-12.5	12.5%	259	78	4	25
PDFS-Vi-25.0	25.0%	222	156	4	25
PDFS-Vi-37.5	37.5%	185	185	4	25
PDFS-Vi-50.0	50.0%	148	312	4	25
